# Supplementary material for: CoFe Alloy-Coupled Mo2C Wrapped by Nitrogen-Doped Carbon as Highly Active Electrocatalysts for Oxygen Reduction/Evolution Reactions
Source: Nanomaterials (Basel). 2023 Jan 29;13(3):543. doi: 10.3390/nano13030543 (PMC9920335; doi:10.3390/nano13030543)
Supplement: Supplementary file 1 [file nanomaterials-13-00543-s001.zip › nanomaterials-2168858-supplementary.pdf]

Supplementary Materials

CoFe Alloy-Coupled Mo_2C Wrapped by Nitrogen-Doped Carbon as Highly Active Electrocatalysts for Oxygen Reduction/Evolution Reactions

Jiahao Xie [1,†], Yu Miao [1,†], Bin Liu [1], Siliang Shao [1], Xu Zhang [1], Zhiyao Sun [1,*], Xiaoqin Xu [1], Yuan Yao [2,*], Chaoyue Hu [1] and Jinlong Zou [1,*]

[1] Key Laboratory of Functional Inorganic Material Chemistry, Ministry of Education of the People's Republic of China, School of Chemistry and Materials Science, Heilongjiang University, Harbin, 150080, China
[2] MIIT Key Laboratory of Critical Materials Technology for New Energy Conversion and Storage, School of Chemistry and Chemical Engineering, Harbin Institute of Technology, Harbin 150080, China
* Correspondence: zhiyao202@126.com (Z.S.); yyuan@hit.edu.cn (Y.Y.); zoujinlong@hlju.edu.cn (J.Z.).
† These authors contributed equally to this work.

1. Experimental Section

1.1. Material Characterizations

Herein, the structure, morphology and compositions of the as-prepared materials were characterized by X-ray diffraction (XRD), X-ray photoelectron spectroscopy (XPS), N_2 adsorption/desorption isotherms, transmission electron microscopy (TEM), scanning electron microscope (SEM) and elemental mappings (energy-dispersive X-ray spectroscopy, EDS). The collection of XRD data was performed using a Rigaku D/max 2500 diffractometer equipped with Cu-Kα radiation (k = 1.5406 Å, 40 kV, 20 mA). The Kratos-AXISUL TRA DLD X-ray photoelectron spectrometer equipped with Al Kα radiation was used to record XPS data. "XPS peak" software was used to fit the XPS data of each element. N2 adsorption and desorption isotherms were determined at 77 k using a micron-scale three-star adsorption analyzer. Brunauer-Emmett-Teller (BET) method and Barrett-Joyner-Halenda (BJH) theory were used to analyze the specific surface area and pore size distribution (PSD), respectively. TEM and high-resolution TEM (HRTEM) images were collected on a JEM-2100 electron microscope (JEOL) at an accelerated voltage of 200 kV. SEM images and elemental mappings were collected using an S-4800 (Hitachi, Japan) scanning electron microscope at an acceleration voltage of 5.0 kV. The contact angle of the sample surface was characterized by the JC20001 contact angle tester.

1.2. Electrochemical Measurements

All of the electrochemical performance tests in this study were tested at room temperature using a typical three-electrode test system on a CHI 760E electrochemical workstation (Chenhua Instrument Co., Ltd., Shanghai, China). The counter electrode was a polished platinum sheet (1 cm^2) electrode, while the reference electrode was a saturated calomel electrode (RHE). The potentials reported in this study were referred to the RHE. 5.0 mg of catalyst was dispersed by ultrasonication in a mixed solution of 50 μL Nafion (Aldrich, 5 wt.%) and 100 μL ethanol for 30 min to prepare the catalyst ink. Then, 5 mL of the suspension (the catalyst was uniformly dispersed therein) was dropped on the surface of the rotating disk electrode (diameter of 4 mm, RDE), and dried under ambient conditions to closely bond the catalyst to the electrode.

1.3. ORR activity Measurements

The ORR performance was tested in 0.1 M KOH solution. The cyclic voltammetry (CV) tests were cycled positively in the potential range from 0 to 1.2 V with a scan rate of 50 mV s^{-1}. Before each CV test, N_2 or O_2 was bubbled to saturate the electrolyte. The

linear sweep voltammetry (LSV) tests were performed at 1600 rpm (50 mV s^{-1}) in the O_2-saturated electrolyte. As previously reported, the Tafel tests were carried out in an O_2-saturated electrolyte at 1600 rpm [86,87]. RDE measurements were obtained by varying the rotation rates (from 400 to 2500 rpm) in the LSV tests. The Koutecky-Levich (K-L) equation was used to analyze the obtained RDE data, and the transferred electron number (n) was obtained using equations 1 and 2 [88].

$$\frac{1}{j} = \frac{1}{j_L} + \frac{1}{j_K} = \frac{1}{B\omega^{1/2}} + \frac{1}{nFkC_O} \quad (1)$$

$$B = 0.62nFC_0D_0^{2/3}\upsilon^{-1/6} \quad (2)$$

where j represented the measured value of the disk current density; jL represented the measured value of the diffusion limit current density; jK represented the measured value of the kinetic-limiting current density; ω was the angular speed of rotating electrode; F represented the Faraday constant (F = 96485 C mol^{-1}); and k was the electron-transfer rate constant; C_0 was the oxygen concentration in the test solution (0.1 M KOH, $C_0 = 1.2 \times 10^{-6}$ mol cm^{-3}); D_0 was the oxygen diffusion coefficient in the test solution (0.1 M KOH, $D_0 = 1.9 \times 10^{-5}$ cm2 s^{-1}), and υ was the kinematic viscosity of 0.1 M KOH (υ = 0.01 cm^2 s^{-1}) [89].

According to previous reports, the tests for electrochemical impedance spectroscopy (EIS) and chronoamperometry (CA) were conducted at 0.8 V (1600 rpm) in an O_2-saturated 0.1 M KOH electrolyte. The accelerated durability tests (ADT) were carried out to investigate the durability of the catalyst after 5000 cycles of CV tests, and LSV results were compared before and after the ADT. RRDE measurements were also carried out in O_2-saturated 0.1 M KOH electrolyte at a scan rate of 5 mV s^{-1} at 1600 rpm. The hydrogen peroxide yield (H_2O_2 (%)) and electron transfer numbers (n) were obtained from the RRDE voltammograms using the following equations 3 and 4 [90,91].

$$n = 4 \times \frac{Id}{Id + (Ir / N)} \quad (3)$$

$$H_2O_2\ \% = 200 \times \frac{Ir / N}{Id + (Ir / N)} \quad (4)$$

where I_d and I_r were the disk current and the ring current, respectively; N was the H_2O_2 collection efficiency of the Pt ring with the value of 0.37.

1.4. OER activity measurements

The OER performance was measured in 1 M KOH solution. The LSV tests were scanned from 1.2 V to 1.8 V at 50 mV s^{-1} by using a glassy carbon electrode as a working electrode at a rotation rate of 1600 rpm. The Tafel, EIS, CA, and ADT tests were conducted in 1 M KOH solution at a rotation rate of 1600 rpm. The electrochemically active surface areas (ECSAs) were evaluated from CV data at the potential range from −0.05 to 0.05 V (vs. RHE) at scanning rates between 2 and 80 mV s^{-1}.4 EIS tests were conducted in the frequency range of 100 kHz to 100 mHz at a 5 mV voltage amplitude. The potential for CA and ADT tests was set at 1.6 V. Turnover frequencies (TOF) value was calculated by assuming that the surface-active Co atoms had undergone the redox reaction just before the onset of OER only involved in OER electrocatalysis. The corresponding equations 5 and 6 are shown as follows:

$$TOF = (j \times NA) / (F \times n \times \Gamma) \quad (5)$$

$$\Gamma = CVarea / (Scan\ rate \times e) \quad (6)$$

where j was the current densit, NA was the values of Avogadro number (6.022 × 1023), n was the electron transfer number (4), F was the Faraday constant (96485 C mol^{-1}), Γ was the Surface concentration, CVarea was the area of redox features considered for the calculation of number of surface-active sites, Scan rate was 0.3 V s^{-1}, and e value was 1.602 × 10^{-19} C [92,93].

Faradaic Efficiency determination had now been accepted as important activity parameter which had to be determined to ensure that the supplied energy was efficiently used for water oxidation than other side reactions. The equation 7 for calculating FE was shown as follows:

$$FE = I_r/(I_d \times N) \quad (7)$$

where I_r and I_d were the ring current and the disk current at a particular potential, respectively; N (0.37) was the current [94,95].

The electrochemically active area (ECSA) can be calculated from the electrical double layer capacitance, which is tested at scan rates of 40, 60, 80, 100, and 120 mV s^{-1} with a potential range from 0.96 to 1.06 V vs. RHE. The formula (8) for calculation of ECSA was shown below.

$$ECSA = C_{dl}/(40\ \mu F\ cm^{-2}\ per\ cm^2) \quad (8)$$

where 40 μF cm^{-2} was a constant to convert capacitance to ECSA. The specific capacitance could be converted into an electrochemical active surface area using the specific capacitance value for a flat standard with 1 cm^2 of real surface area.

2. Results and discussion

Table S1. Chemical compositions (wt. %) of NG-CoFe/Mo_2C-x electrocatalysts obtained from XPS analyses.

Catalysts	C	N	O	Fe	Co	Mo
NG-CoFe/Mo_2C-700	85.72	3.41	6.83	1.23	2.07	0.75
NG-CoFe/Mo_2C-750	90.13	1.53	6.48	1.26	0.28	0.32
NG-CoFe/Mo_2C-800	90.73	1.97	5.27	0.47	1.13	0.43
NG-CoFe/Mo_2C-850	88.76	3.18	4.57	1.67	1.43	0.38
NG-CoFe/Mo_2C-900	91.87	0.72	3.89	1.29	1.92	0.31

Table S2. Textural properties of CoFePBA/PVP, CoFe-C and NG-CoFe/Mo_2C-800 composites.

Samples	S_{BET} (m^2 g^{-1})	Pore volume (cm^3 g^{-1})	Average pore width (nm)
CoFePBA/PVP	145.743	0.258	3.275
CoFe-C	178.574	0.369	3.846
NC-CoFe@Mo_2C-800	322.034	0.764	8.672

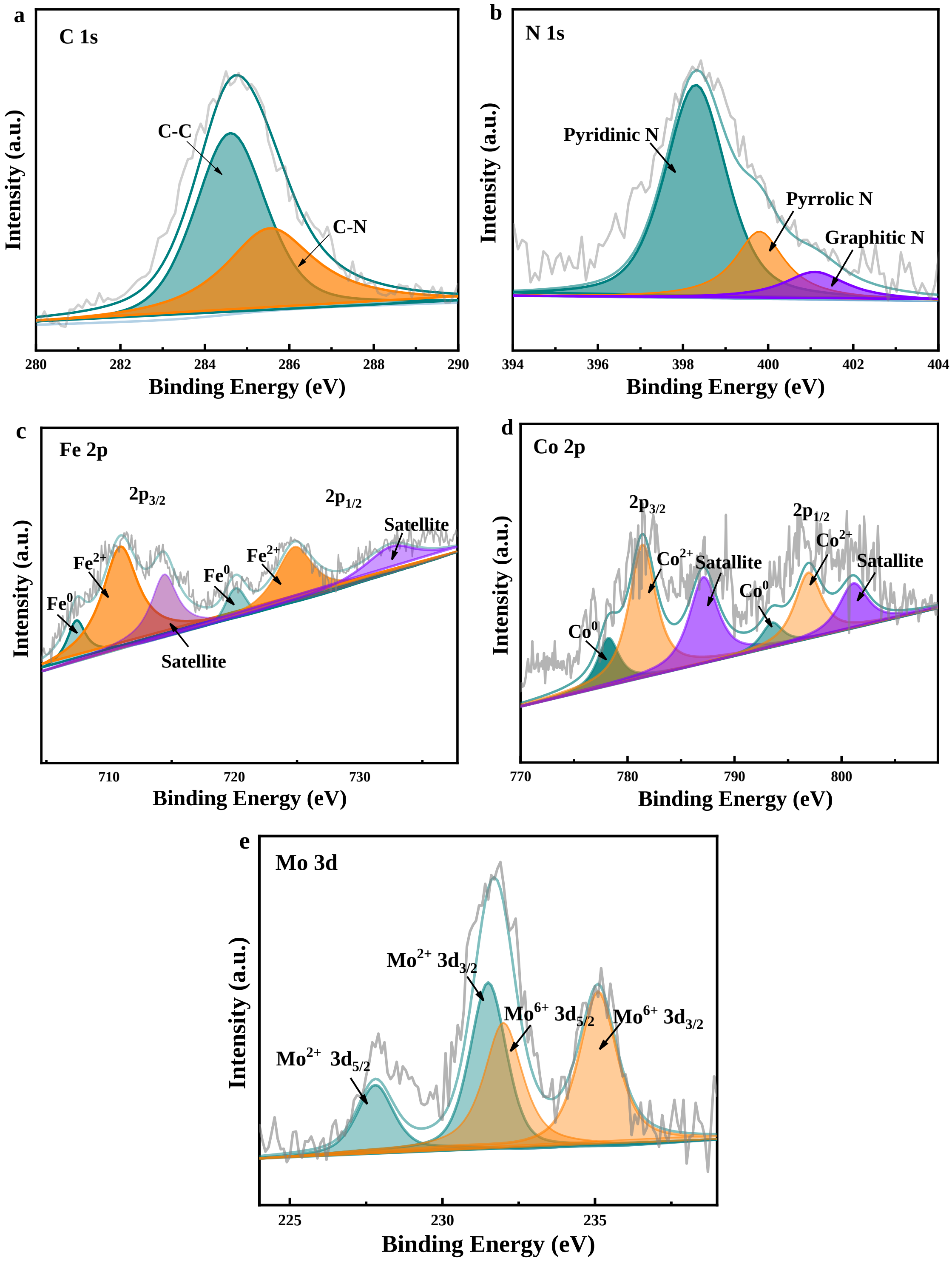

Figure S1. High resolution XPS spectra of C 1s (**a**), N 1s (**b**), Fe 2p (**c**), Co 2p (**d**) and Mo 3d (**e**) for NG-CoFe/Mo_2C-700.

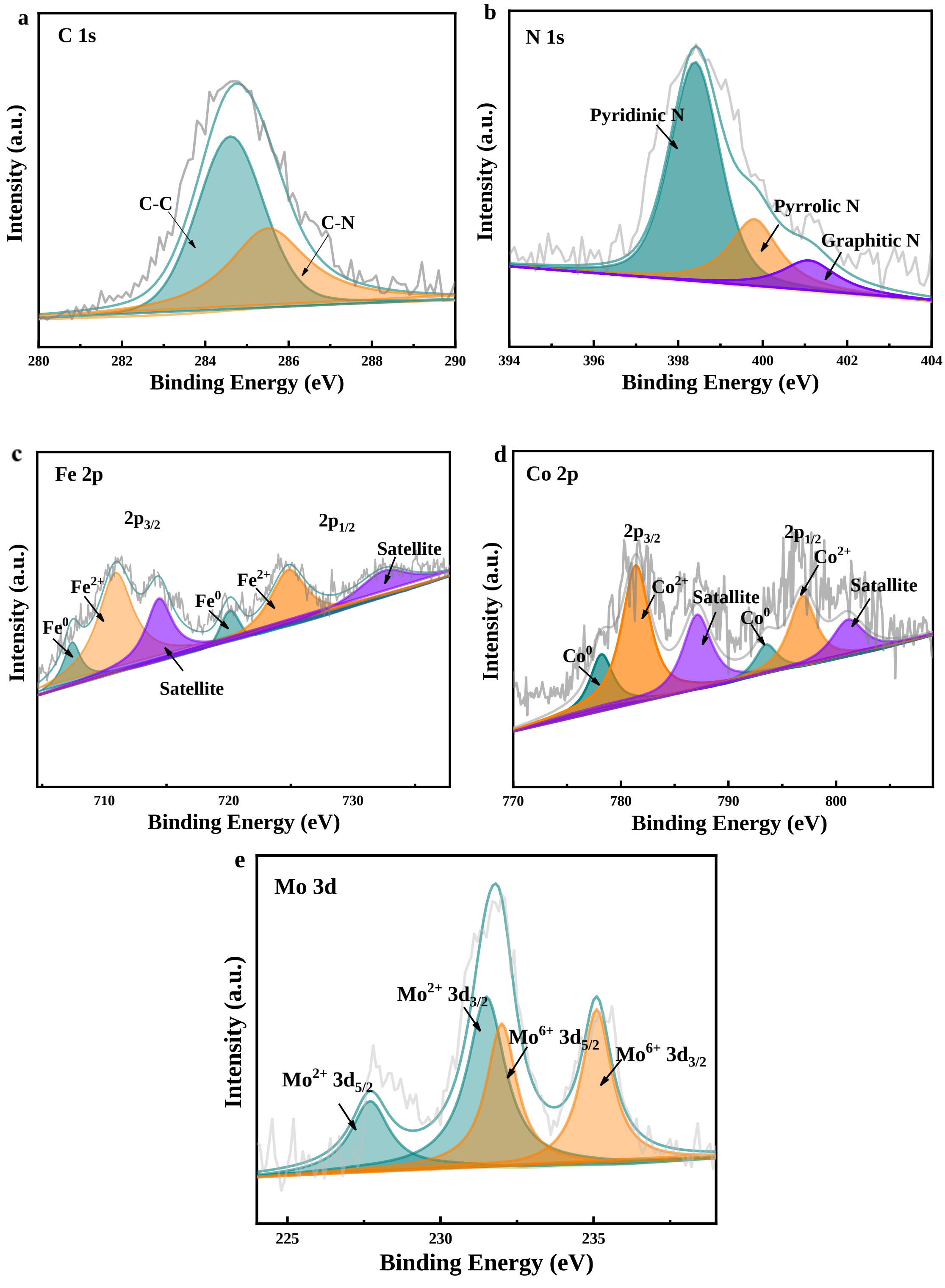

Figure S2. High resolution XPS spectra of C 1s (**a**), N 1s (**b**), Fe 2p (**c**), Co 2p (**d**) and Mo 3d (**e**) for NG-CoFe/Mo_2C-750.

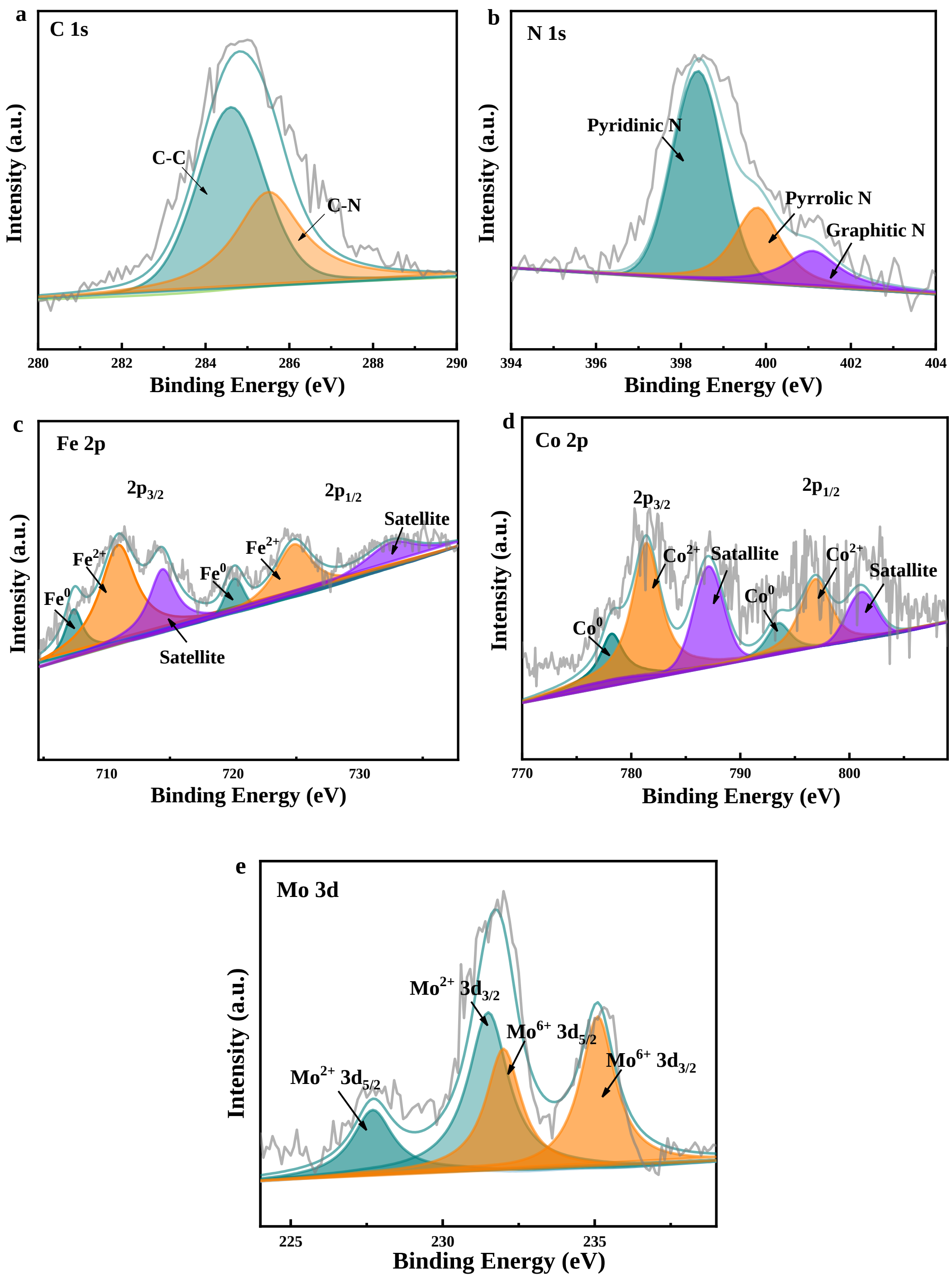

Figure S3. High resolution XPS spectra of C 1s (**a**), N 1s (**b**), Fe 2p (**c**), Co 2p (**d**) and Mo 3d (**e**) for NG-CoFe/Mo_2C-850.

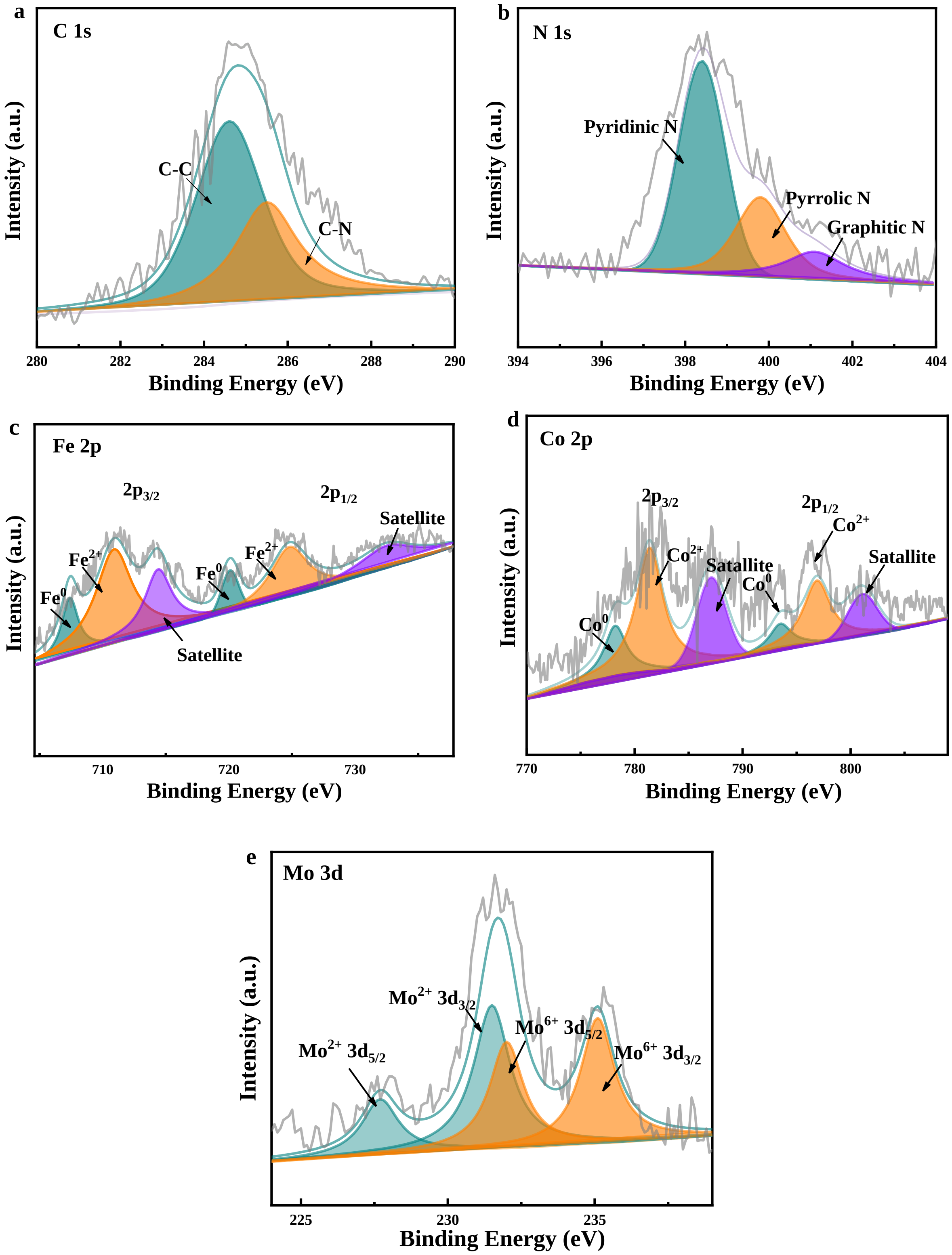

Figure S4. High resolution XPS spectra of C 1s (**a**), N 1s (**b**), Fe 2p (**c**), Co 2p (**d**) and Mo 3d (**e**) for NG-CoFe/Mo_2C-900.

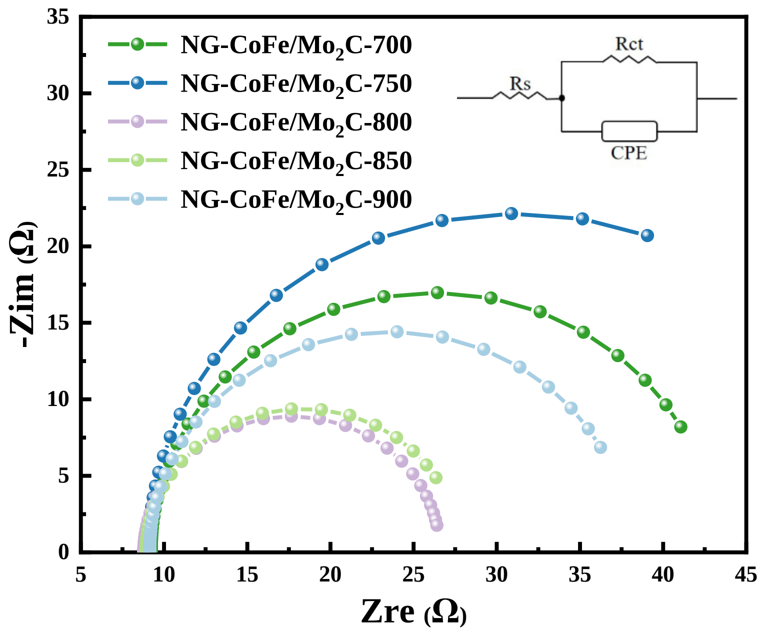

Figure S5. Nyquist curves of NG-CoFe/Mo_2C-x in an O_2-saturated 0.1 M KOH solution at a scan rate of 5 mV s^{-1} with a rotation rate of 1600 rpm for ORR; The inset is the equivalent-circuit diagram.

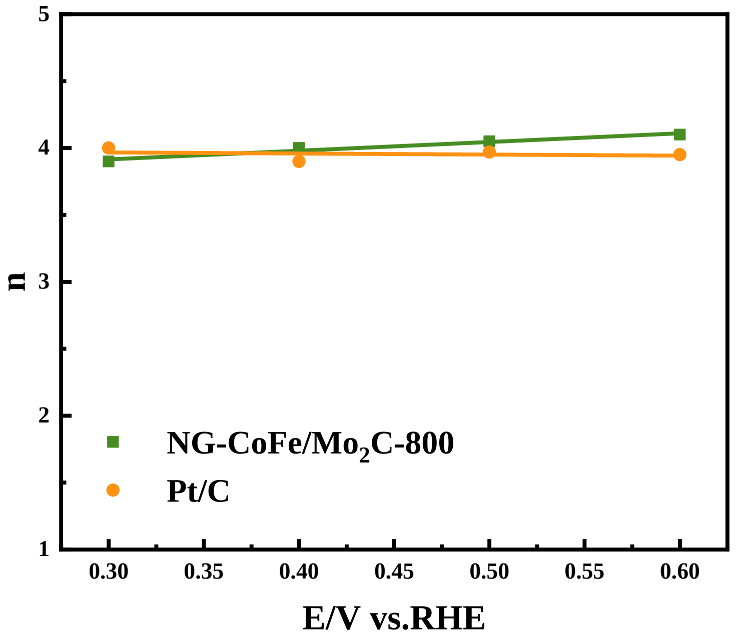

Figure S6. Electron-transfer number n derived from K-L plots at different potentials.

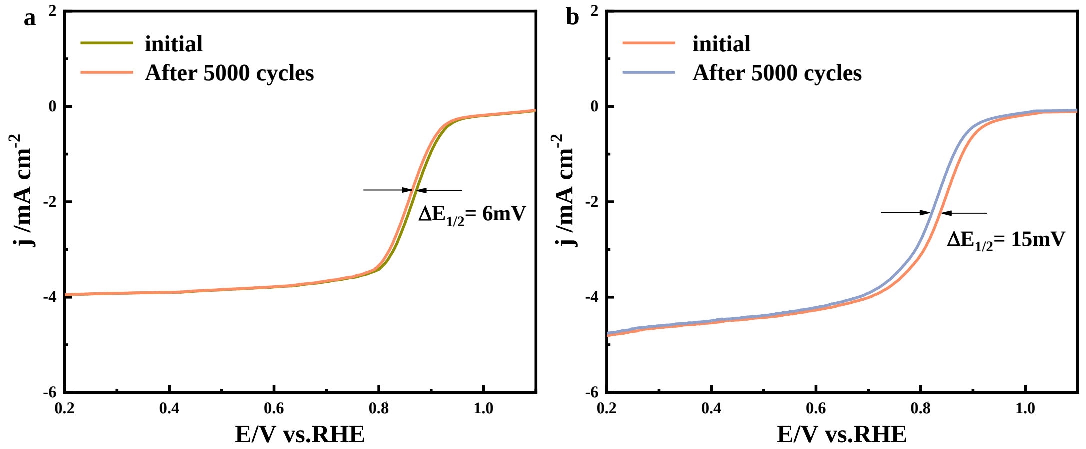

Figure S7. ORR polarization curves of NG-CoFe/Mo_2C-800 (**a**) and Pt/C (**b**) before and after the continuous CV tests in the O_2-saturated 0.1 M KOH at 1600 rpm.

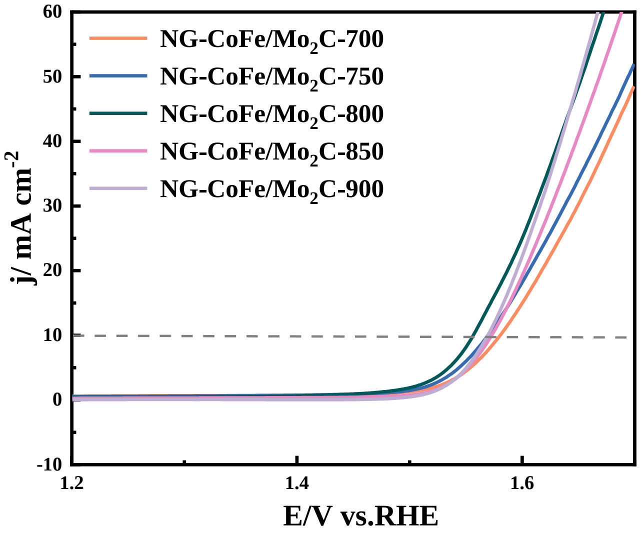

Figure S8. LSV curves of NG-CoFe/Mo_2C-x (x = 700, 750, 800, 850 and 900) in 1 M KOH solution for OER (2 mV s^{-1} and 1600 rpm).

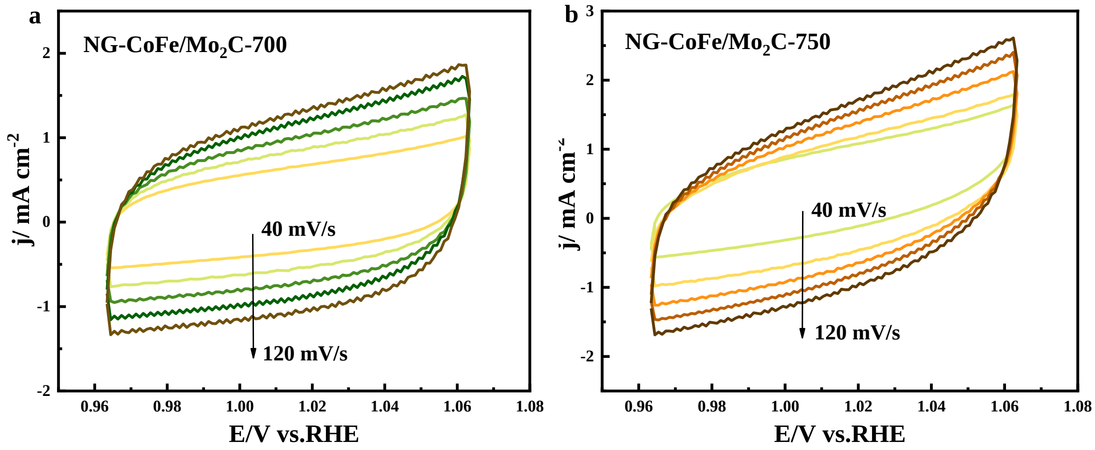

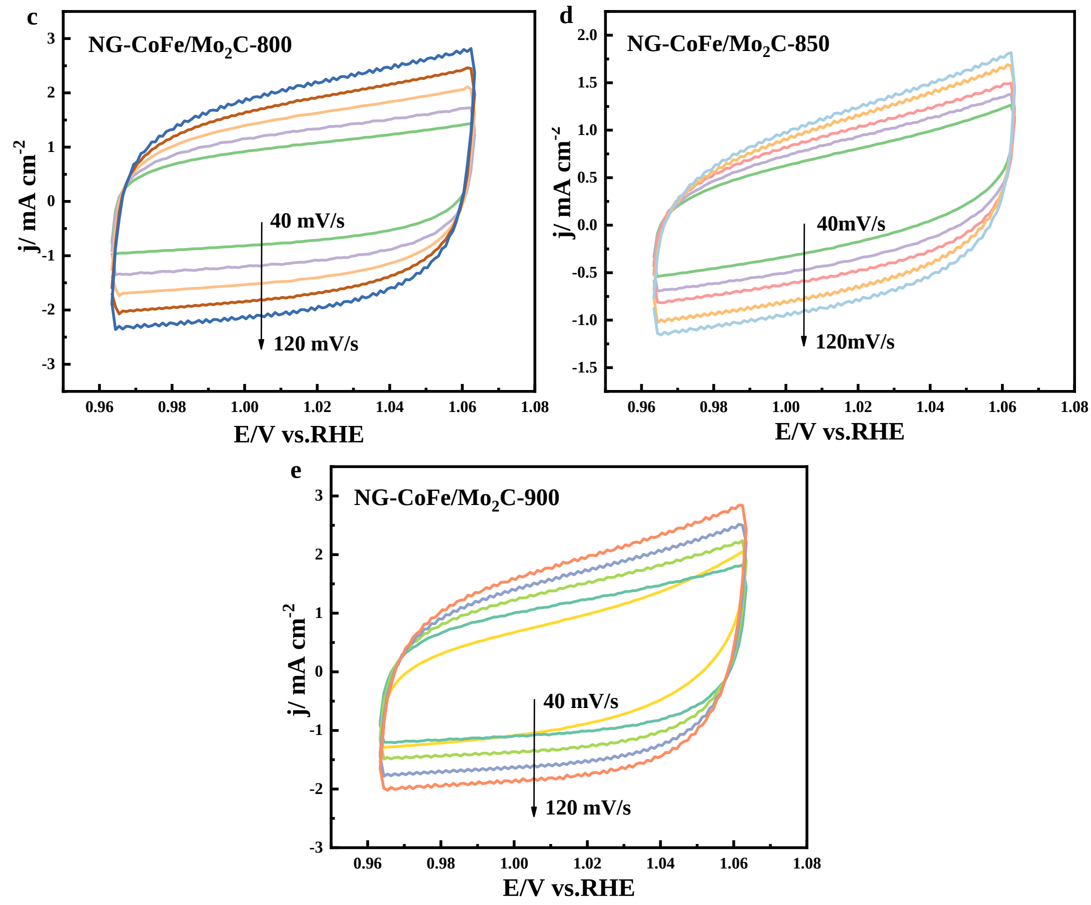

Figure S9. Cyclic voltammograms with various scan rates from 40 to 120 mV s^{-1} of NG-CoFe/Mo_2C-x [(x = 700 (**a**), 750 (**b**), 800 (**c**), 850 (**d**) and 900 (**e**)].

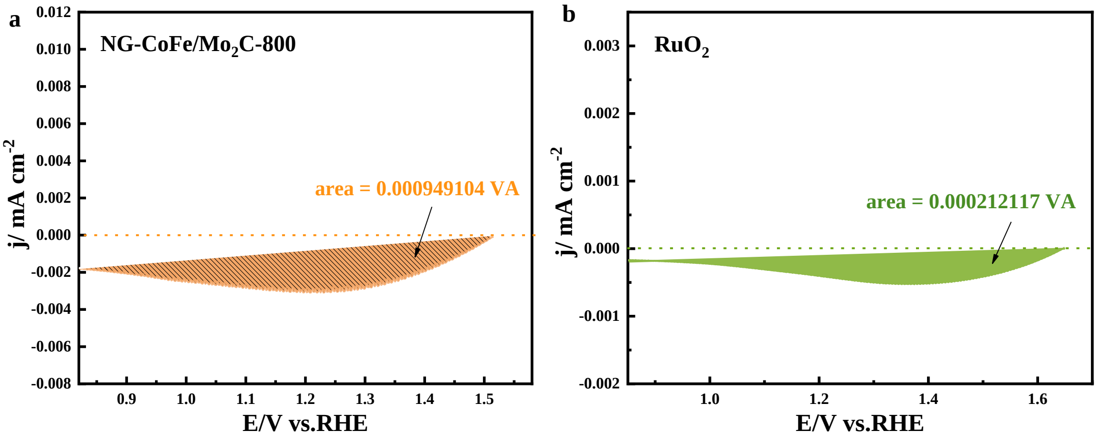

Figure S10. Area of redox features in CV curve of NG-CoFe/Mo_2C-800 (**a**), RuO_2 (**b**) for calculation of the number of surface-active sites.

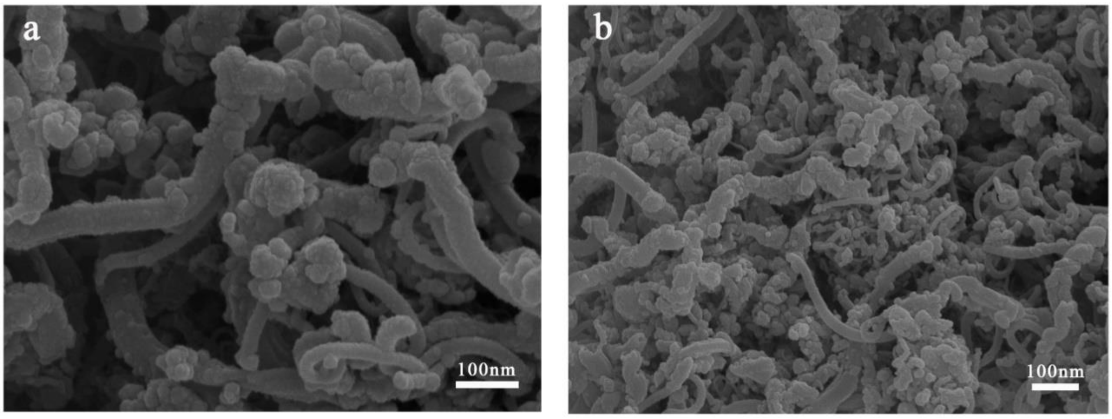

Figure S11. SEM images of NG-CoFe/Mo_2C-800 catalyst after ORR (**a**) and OER (**b**) stability tests.
